# A216 BOWEL URGENCY COMMUNICATION GAP BETWEEN HEALTH CARE PROFESSIONALS AND PATIENTS WITH ULCERATIVE COLITIS IN THE US AND EUROPE: COMMUNICATING NEEDS AND FEATURES OF IBD EXPERIENCES (CONFIDE) SURVEY

**DOI:** 10.1093/jcag/gwac036.216

**Published:** 2023-03-07

**Authors:** S Travis, A P Bleakman, D Rubin, M C Dubinsky, R Panaccione, T Hibi, T H Gibble, C Kayhan, E Flynn, C Sapin, C Atkinson, S Schreiber, J Jones

**Affiliations:** 1 University of Oxford, Oxford, United Kingdom; 2 Eli Lilly and Company, Indianapolis; 3 University of Chicago Medicine Inflammatory Bowel Disease Center, Chicago; 4 Mount Sinai Hospital, New York, United States; 5 University of Calgary, Calgary, Canada; 6 Center for Advanced IBD Research and Treatment, Kitasato University Kitasato Institute Hospital, Tokyo, Japan; 7 Eli Lilly and Company, Indianapolis, India; 8 Adelphi Real World, Bollington, United Kingdom; 9 University Hospital Schleswig-Holstein, Kiel, Germany; 10 Division of Digestive Care and Endoscopy, Department of Medicine, Department of Community Health and Epidemiology, Dalhousie University, Halifax, Canada

## Abstract

**Background:**

The Communicating Needs and Features of IBD Experiences (CONFIDE) study aims to increase understanding of the impact of symptoms on patients with moderate to severe UC and Crohn’s disease and to investigate gaps in communication with healthcare professionals (HCPs) in the United States (US), Europe (EUR), and Japan.

**Purpose:**

This report focuses on patients with moderate to severe UC and HCPs from the US and EUR.

**Method:**

Online, quantitative, cross-sectional surveys of patients with UC and HCPs were conducted in the US and EUR (France, Germany, Italy, Spain, and UK). HCP surveys included physicians and non-physician HCPs responsible for making prescribing decisions. Moderate to severe UC was defined based on treatment, steroid use, and/or hospitalization history. Data collected included perspectives on the experience of patients with UC.

**Result(s):**

A total of 200 US (62% male, mean age 40.4 years) and 556 EUR patients (57% male, mean age 38.9 years), and 200 US and 503 EUR HCPs completed the survey. According to US and EUR patients, the top 3 symptoms currently (past month) experienced were diarrhoea (63% and 50%), bowel urgency (47% and 30%) and increased stool frequency (39% and 30%). Blood in stool was reported as currently experienced by 27% and 24% of US and EUR patients, respectively. Among patients currently experiencing bowel urgency, 47% of US and 27% of EUR patients discuss this symptom at every appointment. Among those who do not discuss bowel urgency at every appointment, 74% and 75% of US and EUR patients would like to discuss this symptom more frequently with their HCP. A total of 30% and 43% of US and EUR patients that ever experienced bowel urgency were not comfortable reporting it to their HCP, with 62% and 58% of these US and EUR patients feeling embarrassed talking about this symptom (Table). HCPs in both the US and EUR ranked diarrhoea (74% and 65%), blood in stool (69% and 65%) and increased stool frequency (38% and 34%) as the top 3 symptoms most reported by patients. According to US and EUR HCPs, the top 4 symptoms proactively discussed in routine appointments were blood in stool (93% and 94%), diarrhoea (90% and 91%), increased stool frequency (82% and 82%) and bowel urgency (76% and 82%). Among HCPs who did not proactively discuss bowel urgency, 47% of US and 40% of EUR HCPs expect patients to bring this up if it is an issue.

**Image:**

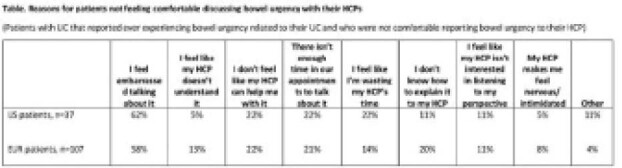

**Conclusion(s):**

Communication gaps were similar between US and EUR patients and HCPs. Bowel urgency is the second-most reported symptom by patients with moderate to severe UC. However, this symptom is not among the HCP-perceived top 3 most reported symptoms. Although a substantial proportion of patients reported a desire to discuss bowel urgency more frequently with their HCP, some patients reported feeling embarrassed talking about it. Many HCPs who do not proactively discuss this symptom expect patients to bring this up. A communication gap was identified and highlights the under-appreciation of bowel urgency as an important symptom of UC.

**Please acknowledge all funding agencies by checking the applicable boxes below:**

Other

**Please indicate your source of funding;:**

Eli Lilly and Company

**Disclosure of Interest:**

S. Travis Grant / Research support from: AbbVie, BUHLMANN Diagnostics, ECCO, Eli Lilly and Company, Ferring Pharmaceuticals, International Organization for the Study of Inflammatory Bowel Disease, Janssen, Merck Sharp & Dohme, Normal Collision Foundation, Pfizer, Procter & Gamble, Schering-Plough, Takeda, UCB Pharma, Vifor Pharma, and Warner Chilcott, A. Bleakman Employee of: Eli Lilly and Company, D. Rubin Grant / Research support from: Takeda, Consultant of: AbbVie, Allergan, AltruBio, American College of Gastroenterology, Arena Pharmaceuticals, Athos Therapeutics, Bellatrix Pharmaceuticals, Boehringer Ingelheim, Bristol Myers Squibb, Celgene/Syneos Health, Cornerstones Health (non-profit), Eli Lilly and Company, Galen/Atlantica, Genentech/Roche, Gilead Sciences, GoDuRn, InDex Pharmaceuticals, Ironwood Pharmaceuticals, Iterative Scopes, Janssen, Materia Prima, Pfizer, Prometheus Therapeutics and Diagnostics, Reistone Biopharma, Takeda, and TechLab, M. Dubinsky Shareholder of: Trellus Health, Grant / Research support from: AbbVie, Janssen, Pfizer, and Prometheus Biosciences, Consultant of: AbbVie, Arena Pharmaceuticals, Boehringer Ingelheim, Bristol Myers Squibb, Celgene, Eli Lilly and Company, F. Hoffmann-La Roche, Genentech, Gilead Sciences, Janssen, Pfizer, Prometheus Therapeutics and Diagnostics, Takeda, and UCB Pharma, R. Panaccione Grant / Research support from: AbbVie, Ferring Pharmaceuticals, Janssen, Pfizer, and Takeda, Consultant of: Abbott, AbbVie, Alimentiv, Amgen, Arena Pharmaceuticals, AstraZeneca, Biogen, Boehringer Ingelheim, Bristol Myers Squibb, Celgene, Celltrion, Cosmo Pharmaceuticals, Eisai, Elan Pharma, Eli Lilly and Company, Ferring Pharmaceuticals, Galapagos NV, Genentech, Gilead Sciences, GlaxoSmithKline, Janssen, Merck, Mylan, Oppilan Pharma, Pandion Therapeutics, Pfizer, Progenity, Protagonist Therapeutics, Roche, Sandoz, Satisfai Health, Shire, Sublimity Therapeutics, Takeda, Theravance Biopharma, and UCB Pharma, T. Hibi Grant / Research support from: AbbVie, Activaid, Alfresa Pharma, Bristol Myers Squibb, Eli Lilly Japan K.K., Ferring Pharmaceuticals, Gilead Sciences, Janssen Pharmaceutical K.K., JMDC, Nippon Kayaku, Mochida Pharmaceutical, Pfizer Japan, and Takeda, Consultant of: AbbVie, Apo Plus Station, Bristol Myers Squibb, Celltrion, EA Pharma, Eli Lilly and Company, Gilead Sciences, Janssen, Kyorin, Mitsubishi Tanabe Pharma, Nichi-Iko Pharmaceutical, Pfizer, Takeda, and Zeria Pharmaceutical, Speakers bureau of: AbbVie, Aspen Japan K.K., Ferring Pharmaceuticals, Gilead Sciences, Janssen, JIMRO, Mitsubishi Tanabe Pharma, Mochida Pharmaceutical, Pfizer, and Takeda, T. Gibble Employee of: Eli Lilly and Company, C. Kayhan Employee of: Eli Lilly and Company, E. Flynn Employee of: Eli Lilly and Company, C. Sapin Employee of: Eli Lilly and Company, C. Atkinson Consultant of: Eli Lilly and Company in connection with the development of this publication, Employee of: Adelphi Real World, S. Schreiber Grant / Research support from: personal fees and/or travel support from: AbbVie, Amgen, Arena Pharmaceuticals, Biogen, Bristol Myers Squibb, Celgene, Celltrion, Eli Lilly and Company, Dr. Falk Pharma, Ferring Pharmaceuticals, Fresenius Kabi, Galapagos NV, Gilead Sciences, I-MAB Biopharma, Janssen, Merck Sharp & Dohme, Mylan, Novartis, Pfizer, Protagonist Therapeutics, Provention Bio, Roche, Sandoz/Hexal, Shire, Takeda, Theravance Biopharma, and UCB Pharma, J. Jones: None Declared

